# Establishment and characterization of clear cell renal cell carcinoma cell lines with different metastatic potential from Chinese patients

**DOI:** 10.1186/1475-2867-13-20

**Published:** 2013-02-26

**Authors:** Xiaojie Tan, Songqin He, Yifang Han, Yongwei Yu, Jianru Xiao, Danfeng Xu, Guoping Wang, Yan Du, Wenjun Chang, Jianhua Yin, Tong Su, Jianguo Hou, Guangwen Cao

**Affiliations:** 1 Department of Epidemiology, Second Military Medical University, 800 Xiangyin Rd, Shanghai 200433, China; 2 Department of Pathology, the 1st Affiliated Hospital, Second Military Medical University, Shanghai, China; 3 Department of Orthopaedics Surgery, the 2nd Affiliated Hospital, Second Military Medical University, Shanghai, China; 4 Department of Urology, the 2nd Affiliated Hospital, Second Military Medical University, Shanghai, China; 5 Department of Urology, the 1st Affiliated Hospital, Second Military Medical University, Shanghai, China

**Keywords:** Renal cell carcinoma, Clear cell, Cell line, Metastasis, Chinese

## Abstract

**Abstracts:**

**Background**

Clear cell renal cell carcinoma (ccRCC) cell lines with distinct metastatic potential are essential to study the mechanism of ccRCC metastasis. However, none of them originated from Chinese.

**Methods**

Primary cell cultures were performed using a primary tumor of a 49-year-old male ccRCC patient and a metastatic tumor of a 62-year-old male patient who had received nephrectomy to excise primary ccRCC 10 years ago. Cell growth, microstructure, cytogenetics, cytometry, expression of metastasis-associated molecules, tumorigenesis and metastasis were subsequently characterized.

**Results**

Two successive cell lines named NRCC from the primary ccRCC and MRCC from the metastatic ccRCC were established, respectively. Compared to NRCC, MRCC exhibited stronger anchorage-independent growth and invasion potentials and contained more glycogen granules in the cytoplasm. Gains of chromosomes and some translocations were the major chromosomal aberrations in both cell strains. CD24 expression was more frequent in MRCC than in NRCC and the same was true for CD56. The transcriptional levels of *TNF*α*, IL-6, VEGF, HIF2*α*, MMP2*, and *RhoC* were significantly higher in MRCC than in NRCC. Cytosolic IκBα protein was more degraded in MRCC than in NRCC following TNFα treatment. Both cell lines had strong tumorigenicity in athymic nude mice. However, MRCC had strong potential in generating metastasis to lung and hemorrhagic ascites than NRCC following orthotopic transplantations.

**Conclusions**

Cancer cells isolated from metastatic ccRCC have more malignant and metastatic potential than those from the primary tumor from the patients who shared the similar race background. Establishment of MRCC and NRCC may provide suitable models with which to investigate molecular mechanisms of ccRCC metastasis.

## Background

The incidence of renal cell carcinoma (RCC) varies substantially worldwide. The rates are generally high in Europe and North America while low in Asia and South America [1]. RCC is a pathologically heterogeneous disease and can be subdivided into clear, papillary, granular, spindle, and mixed cell subtypes based on cytoplasmic features. Clear cell RCC (ccRCC) is the most common type (70%-80%) and accounts for most cases of metastatic disease. Metastatic RCC is a highly fatal disease, which accounts for about a third of the patients at initial presentation. Approximately 10% to 28% of RCC develop a local recurrence or distant metastasis after curative nephrectomy [2]. Metastatic RCC is resistant to chemotherapy and radiotherapy but responds to tyrosine kinase inhibitors and interleukin-2-based immunotherapy [3,4].

Asian and non-Asian populations exhibit big differences in the incidence of RCC, environmental and genetic risk factors, and even adverse effects of the treatment with sorafenib and sunitinib [1,5,6]. Genetic background should be important in exploring the mechanism of renal carcinogenesis and developing therapeutic option. RCC cell lines with diverse metastatic potential are essential in understanding RCC biology and the mechanism of metastasis and also beneficial for identification of therapeutic approaches to improve the prognosis. Several RCC cell lines have been isolated and characterized [7,8]. However, ccRCC cell lines with characterized metastatic potential are very rare and none of them were from Chinese.

In this study, we characterized the two ccRCC cell lines with different metastatic potential. One was derived from primary ccRCC and the other was from a metastatic ccRCC. The comparable cell lines can be used for exploration of metastasis mechanism, selection of therapeutic compounds, and development of ccRCC vaccines. To our knowledge, this is the first report of the establishment of ccRCC cell lines from Chinese patients.

## Results

### Establishment of ccRCC cell lines

We succeeded in maintaining the cultured cells from clinical specimens of the two patients and culturing them for more than 100 generations in DMEM with 10% FCS. The cells from both patients were proven to have strong tumorigenicity in nude mice. Primary cultures were also made using the subcutaneous and orthotropic tumors derived from the two cell lines. The successive ccRCC cell lines derived from the metastatic site of the patient (No.375771) and from the primary ccRCC tissue of the patient (No.378570) were termed as MRCC and NRCC, respectively. MRCC and NRCC cells were reserved in Chinese Center for Type Culture Collection (CCTCC) with store numbers CCTCC-C200909 and CCTCC-C200910, respectively. The following assays were carried out using the two cell lines of 50–60 generations.

### Morphology of MRCC and NRCC

**Figure 1:**
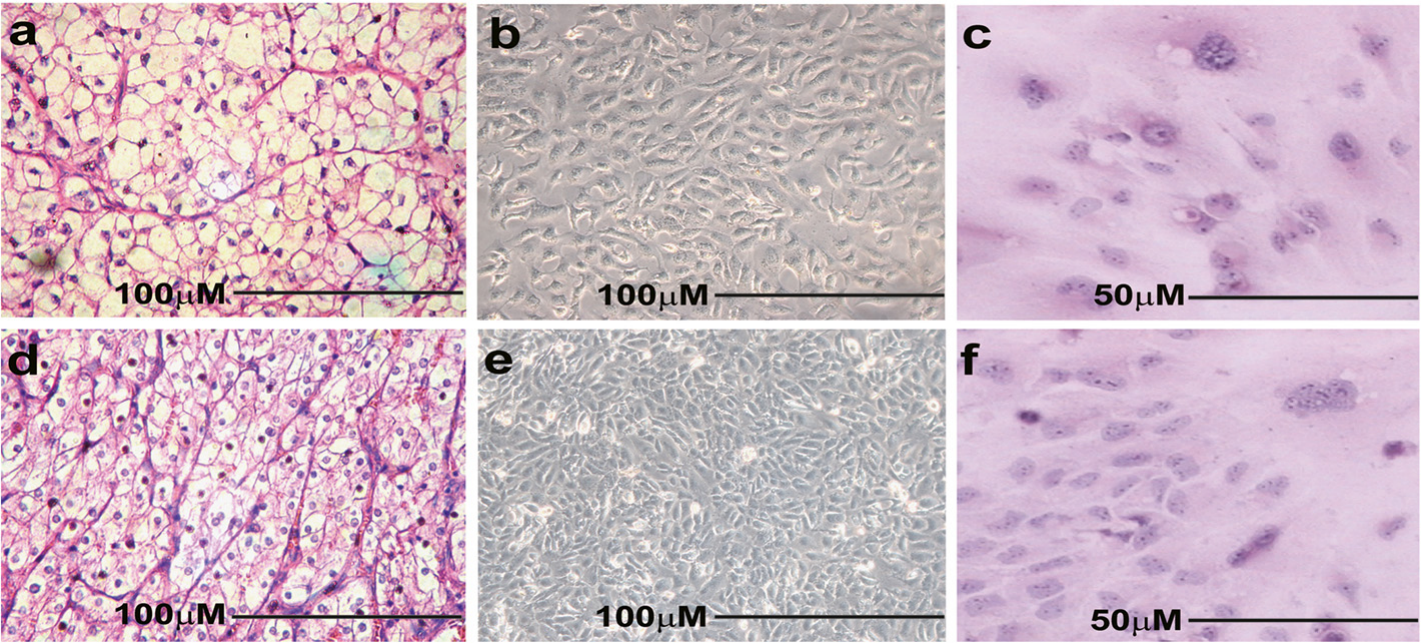
**Morphology of MRCC and NRCC cells. **(**a**-**c**) MRCC; (**d**-**f**) NRCC; (**a**, **d**) the corresponding tumors, H&E staining; (**b**, **e**) cell culture; (**c**, **f**) cell culture, H&E staining.

**Figure 2:**
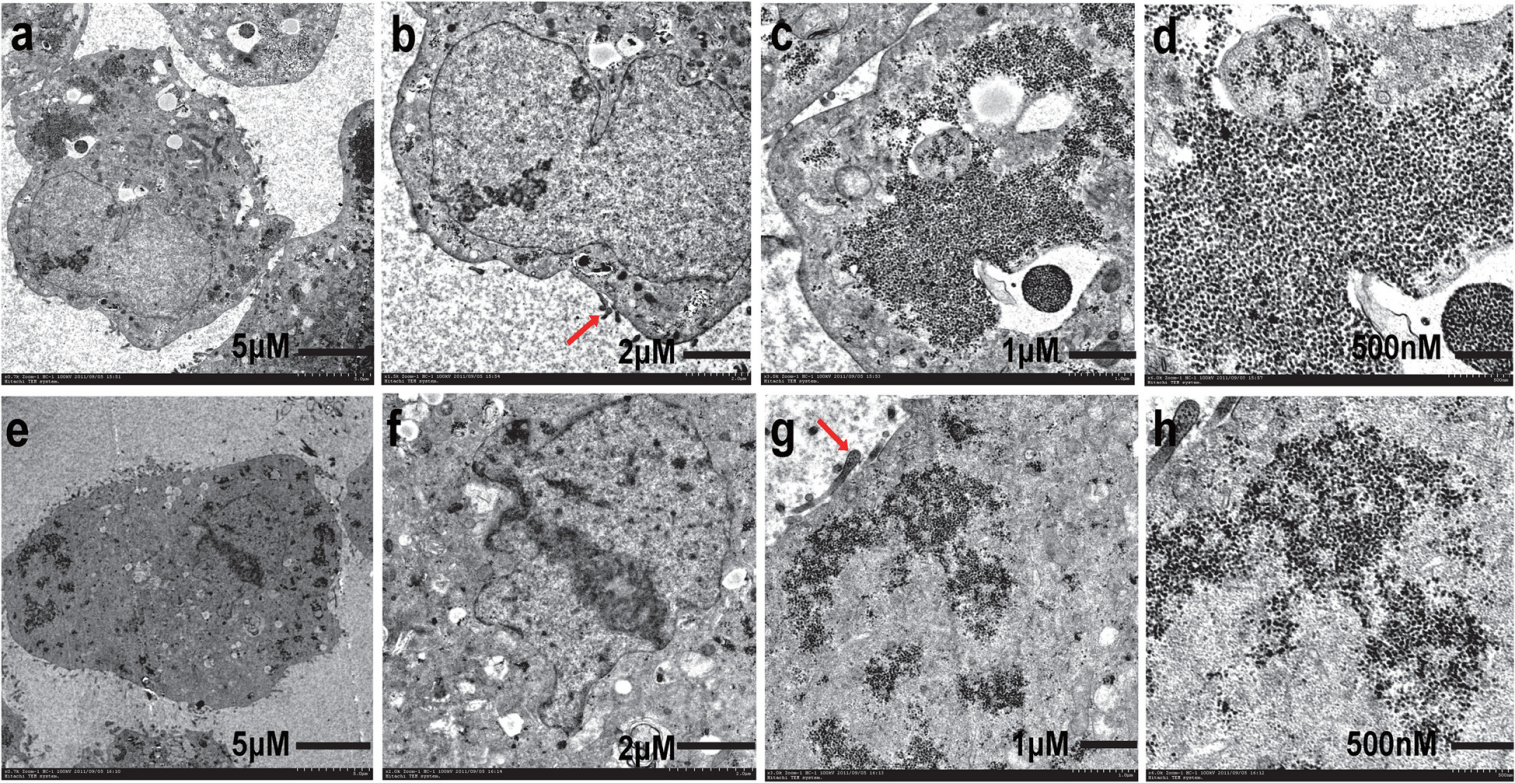
**EM findings. **(**a**-**d**) MRCC; (**e**-**h**) NRCC; (**a**, **e**) the entire cell; (**b**) nucleus and microvilli (red arrow); (**c**) glycogen granules and mitochondria; (**d**, **h**) glycogen granules accumulated; (**f**) nucleus and mitochondria; (**g**) glycogen granules and microvilli (red arrow).

Figure 1 shows the morphology of the cell lines and parental tissues. The cell lines had clear cytoplasm, round to oval nuclei with one or two nucleoli, and high nuclear-to-cytoplasmic ratio. NRCC cells were typically epithelial-like and spindle-shaped and proliferated in a pavement-like cell arrangement with distinct border. As compared to NRCC, MRCC cells were relatively larger in size with irregular shapes and a lack of distinct border. MRCC proliferated in a pavement-like cell arrangement with a lack of contact inhibition. The entire cells of MRCC and NRCC were shown as Figure 2a and 2e, respectively. The two cell lines were of epithelial cell origin as microvilli detected on their cell surfaces (Figure 2b and 2g). Ultrastructurally, the cell lines displayed characteristic filmy cytoplasm containing abundant glycogen granules, lipid droplets, and poorly developed mitochondria (Figure 2c and 2f) and nuclei with prominent nucleoli, which was consistent with the previous EM findings of ccRCC [8,9]. Interestingly, the glycogen granules were more clustered in MRCC than in NRCC (Figure 2d and 2h).

### Growth kinetics and invasion ability

The doubling times of NRCC cells and MRCC cells were 35.5 h and 55.2 h, respectively. However, MRCC cells showed stronger anchorage-independent growth than NRCC cells. Colony formation rates of MRCC cells and NRCC cells in the agarose were 45.3% ± 4.9% and 31.7% ± 4.5% (*p* < 0.05), respectively. In the invasion assay, it was found that the invasive capacity of MRCC cells surpassed that of NRCC cells (average invasion rates: 43.6% ± 5.8% *vs.* 30.2% ± 4.6%, *p* < 0.001). These results indicated that MRCC cells could be more invasive than NRCC cells.

### Cytogenetic characteristics

**Figure 3:**
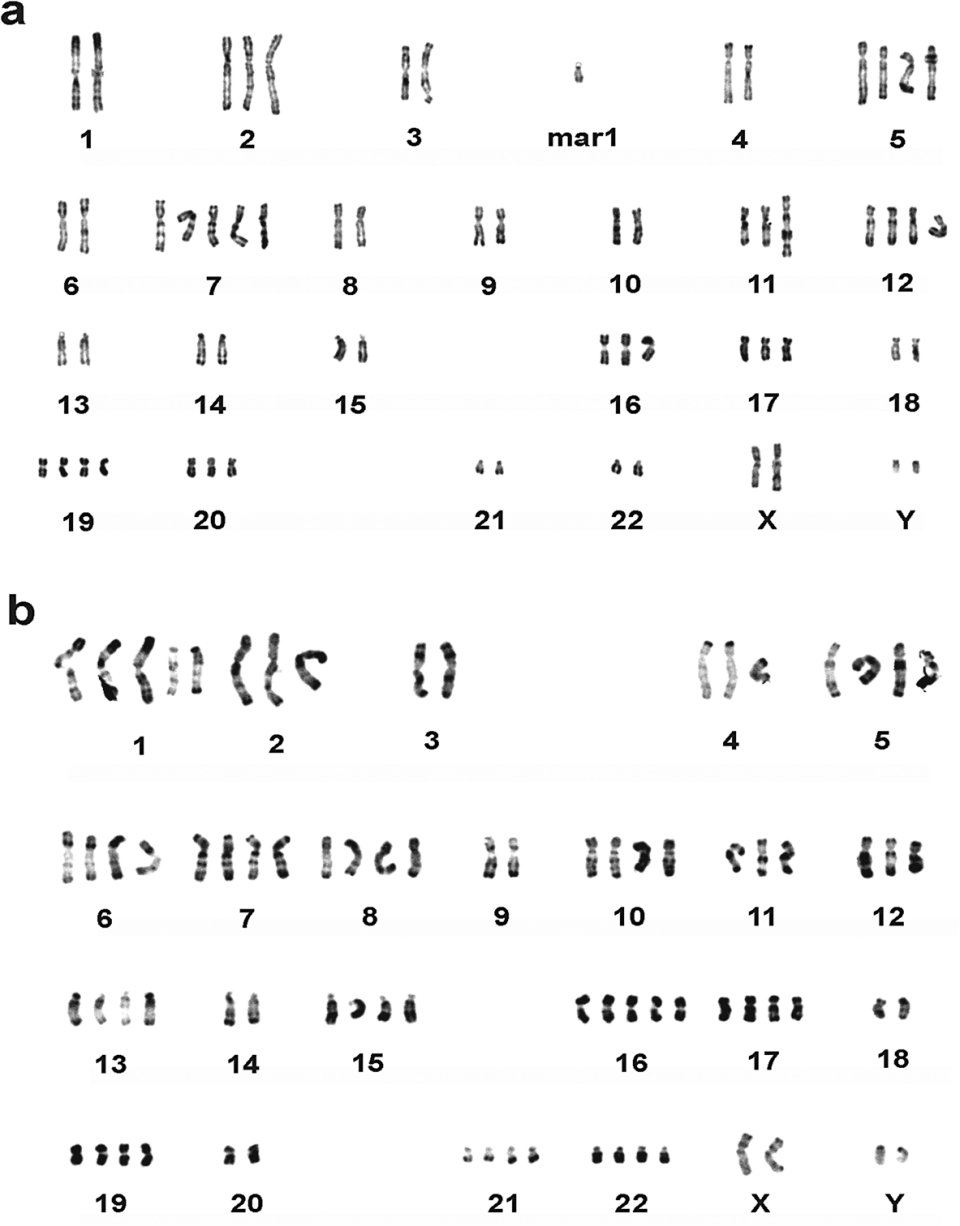
**Representative G-banding karyotypes of MRCC and NRCC. **(**a**) MRCC; (**b**) NRCC. mar1, a marker chromosome.

The number of chromosomes in MRCC cells ranged from 45 to 68 with a modal number of 66; while that in NRCC cells ranged from 53 to 86 with a modal number of 82. Gains of chromosomes, rather than loss of chromosomes, were the major abnormalities in chromosome number. Table 1 shows the gain of chromosomes in the two cell lines. Two significant chromosomal translocations, der(6)t(3qter → q12::6q11 → pter) and der(11)t(11pter → q23::1q23 → qter) were identified in 80.0% and 96.7% of MRCC cells, respectively. Furthermore, a marker chromosome (mar1) was observed in 93.3% of MRCC cells. One deletion, del(4)(pter → q25:) and a translocation, der(1)t(1qter → q11::13q11 → qter) were found in 93.3% and 36.7% of NRCC cells, respectively. Figure 3 presents typical karyotype of each cell line for chromosomal abnormality in both number and structure.

**Table 1:** Gains of chromosomes in MRCC and NRCC cell lines

**Chromosome No.**	**MRCC**		**NRCC**	
	**Gain**	**Frequency (%, n/N)** †	**Gain**	**Frequency (%, n/N)** †
1	-	-	1-4	100.0 (30/30)
2	1	63.6 (19/30)	1-2	100.0 (30/30)
4	-	-	1-2	90.0 (27/30)
5	1-2	100.0 (30/30)	1-3	96.7 (29/30)
6	1	70.0 (21/30)	1-2	96.7 (29/30)
7	1-3	93.3 (28/30)	1-3	93.3 (28/30)
8	-	-	1-2	90.0 (27/30)
10	-	-	1-3	90.0 (27/30)
11	1	96.7 (29/30)	1-3	93.3 (28/30)
12	1-2	93.3 (28/30)	1-2	93.3 (28/30)
13	-	-	1-2	83.3 (25/30)
15	-	-	1-3	96.7 (29/30)
16	1	66.7 (20/30)	2-4	93.3 (28/30)
17	1-2	80.0 (24/30)	1-3	96.7 (29/30)
19	1-2	73.3 (22/30)	1-4	96.7 (29/30)
20	1-3	96.7 (29/30)	-	-
21	-	-	1-3	90.0 (27/30)
22	-	-	1-2	96.7 (29/30)

† n, the number of metaphases with gain(s) of chromosome(s); N, total number of metaphases observed.

### Expression of cell markers

**Figure 4:**
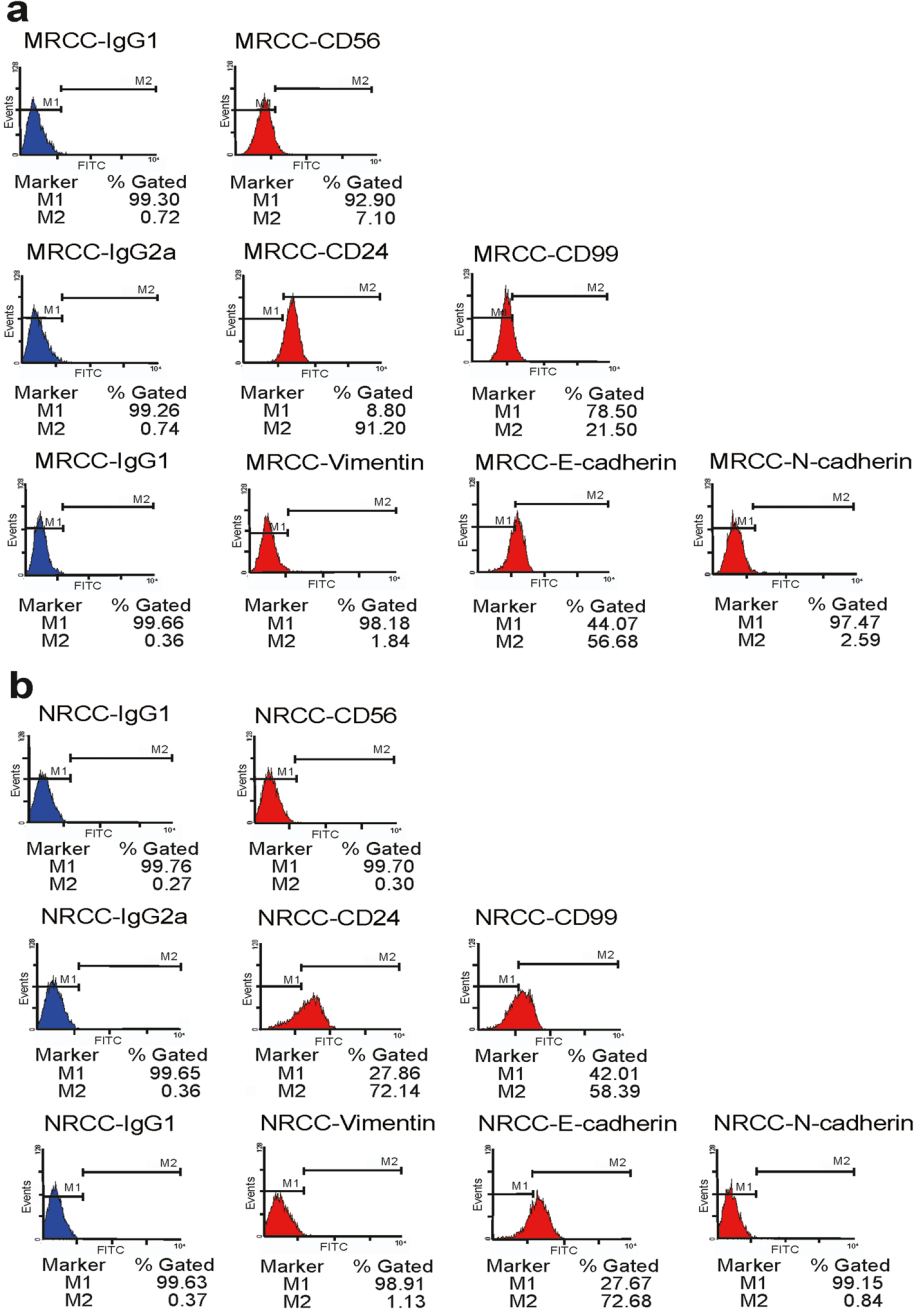
**Flow cytometry for the analyses of CD56, CD24, CD99, vimentin, E- cadherin, N-cadherin expression in MRCC and NRCC. **(**a**) MRCC; (**b**) NRCC.

Taking into account a previous report about stem-like cell markers of ccRCC [10], we examined the expression of CD105, CD133, CD44, CD24, CD56, CD99, and CD74 on MRCC and NRCC cells by cytometry. It was found that both cell lines were positive for CD44 but negative for CD133, CD105, and CD74. The positive rate of CD24 was higher in MRCC cells (Figure 4a) than in NRCC cells (Figure 4b) and the same was true for CD56. However, the positive rate of CD99 was higher in NRCC cells than in MRCC cells. We also examined the expression of epithelial and mesenchymal markers. In contrast to N-cadherin expression, E-cadherin expression was less frequent in MRCC cells than in NRCC cells. The expression of vimentin was a little higher in MRCC cells than in NRCC cells. These data indicated that NRCC cells displayed more epithelial characteristics, while MRCC cells tended to be more mesenchymal-like.

### Cell cycle analysis

**Figure 5:**
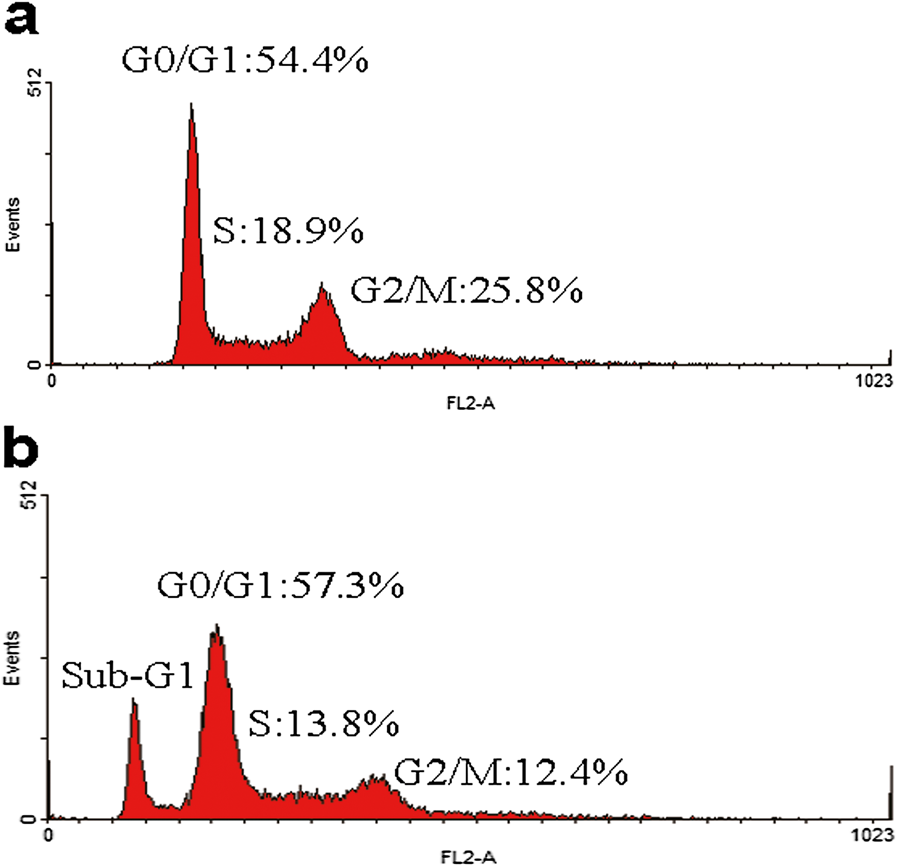
**DNA content of the ccRCC cell lines by flow cytometry. **(**a**) MRCC; (**b**) NRCC.

Cell cycle analysis showed that 18.9% of MRCC cells were in S phase; while 13.8% of NRCC were in S phase (Figure 5). Interestingly, a typical sub-G1 peak was frequently detected in NRCC cells, rather than in MRCC cells, indicating a possible apoptosis undertaken in NRCC cells.

### Expression of metastasis-associated molecules

**Figure 6:**
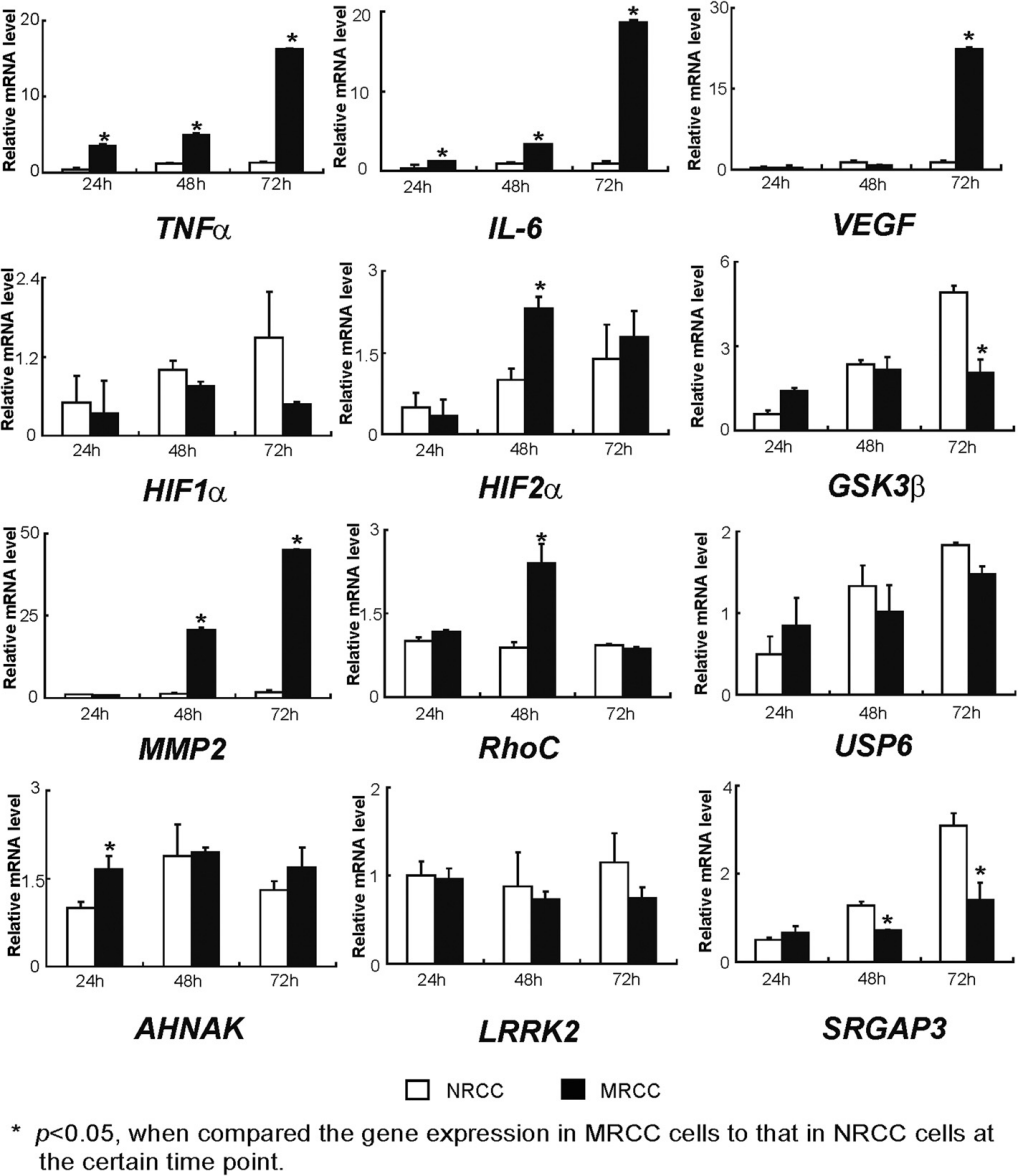
Relative mRNA levels of genes of interest in MRCC and NRCC.

**Figure 7:**
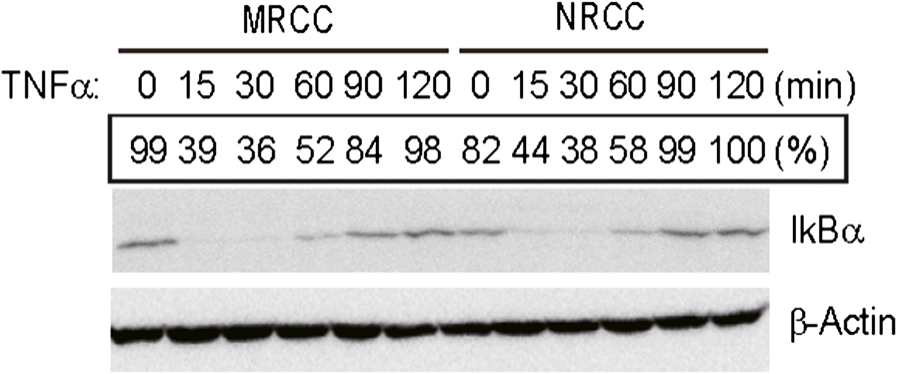
Western blotting for the detection of IkBα degradation following TNFα treatment.

Since the doubling times of the two cell lines were different, the transcription of i*nterleukin-6 (IL-6), tumor necrosis factor-α (TNFα), vascular endothelial growth factor (VEGF), matrix metalloproteinase-2 (MMP2), hypoxia-inducible factor (HIF) 1α, HIF2α, glycogen synthase kinase 3 beta (GSK3ß), RhoC, Ubiquitin specific peptidase 6(USP6), AHNAK nucleoprotein (AHNAK), Leucine-rich repeat kinase 2 (LRRK2), SLIT-ROBO Rho GTPase activating protein 3 (SRGAP3)* in MRCC cells and NRCC cells were examined at 24 h, 48 h and 72 h, respectively, after cell split. The expression patterns of the examined genes are shown in Figure 6. In general, the expression of the most genes at 24 h culture wasn’t significant different between MRCC and NRCC cells, except that the expression of *TNFa, IL-6*, and *AHNAK* was significantly higher in MRCC cells than in NRCC cells (*p* < 0.05 for each). Together with cell invasion assay performed at 24 h after cell split, the three genes were supposed to be involved in cell mobility and invasion. The expression of *LRRK2* and *USP6* was not significantly different between MRCC and NRCC cells. The expression of *SRGAP3* was significantly higher in NRCC than in MRCC cells at 48 h and 72 h after cell spilt (*p* < 0.05). The levels of *TNFα*, IL-6, *VEGF*, and *MMP2* were higher in MRCC cells than in NRCC cells at 72 h after cell split (*p* < 0.05 for each). The same trend was observed for HIF2α expression at 48 h after cell split (*p* < 0.05). However, the expression of HIF1α was higher in NRCC cells than in MRCC cells 72 h after cell split but the difference did not reach a significant level. The expression of *GSK3β* was lower in MRCC cells than in NRCC cells at 72 h after cell split (*p* < 0.05). Furthermore, the *RhoC* expression was higher in MRCC cells than NRCC cells at 48 h after cell split (*p* < 0.05). Western blot indicated that cytosolic IκBα protein was more degraded in MRCC cells than in NRCC cells within 60 min after the *in vitro* treatment with TNFα (Figure 7), indicating nuclear factor-kappa B (NF-κB) signaling pathway is more active in MRCC than in NRCC cells.

### Metastatic potential of MRCC and NRCC in nude mice

Subcutaneous transplantation of MRCC cells or NRCC cells generated tumors in nude mice within two weeks. No lung metastasis was detected following the first round of surgical orthotopic implantation (SOI) with MRCC and NRCC tumors. However, ccRCC metastasized to lung was frequently detected in the mice since the second cycle of SOI with MRCC tumors, and the incidence of lung metastasis was nearly 100%. Furthermore, the metastasis to lymph nodes near ventral aorta and hemorrhagic ascites were frequently evident in the SOI mice transplanted with MRCC tumors. With the increase of the cycles of the transplantation with MRCC, the duration of orthotopic tumor formation to about 10 mm in diameter became shorter and the incidences of hemorrhagic ascites and cachexy became higher (Table 2). We isolated and established the metastatic cell strain from pulmonary tumor mass, named MRCC-L. MRCC-L looked smaller in size and grew faster *in vitro* than their parental MRCC. Orthotopic transplantation of NRCC also generated tumor in all mice. However, metastasis was not observed at the first three rounds of the transplantation with NRCC cells. These data suggested that MRCC had higher malignant and metastatic potential than NRCC.

**Table 2:** Tumorigenicity and metastasis of the two cell lines following orthotopic transplantation in nude mice

**Round No.**	**Tumor source**	**Incidence of tumorigenicity (a/b) **†	**Enlarged lymph nodes near the ventral aorta**	**Pulmonary metastasis (a/b) **†	**Hemorrhagic ascites (a/b) **†	**Cachexy (a/b)** †
**MRCC**						
1	Primary	5/5	No	0/5	0/5	0/5
2	Primary	5/5	Yes	4/5	4/5	2/5
3	Metastasis	5/5	Yes	4/5	4/5	3/5
4	Metastasis	5/5	Yes	4/5	2/5	3/5
5	Metastasis	5/5	Yes	5/5	3/5	5/5
6	Metastasis	5/5	Yes	5/5	3/5	5/5
**NRCC**						
1	Primary	4/5	No	0/5	0/5	0/5
2	Primary	5/5	No	0/5	0/5	0/5
3	Primary	5/5	No	0/5	0/5	0/5

† a, the number of mice with the event of interest (tumorigenicity, metastasis, hemorrhagic ascites or cachexy);

b, total number of mice orthotopically transplanted with the minced tumor masses.

## Discussion

In this study, we successfully established two ccRCC cell lines from two Chinese patients with ccRCC. Up to now, the two cell lines have been maintained in our laboratory for 6 years and cultured for more than 100 passages. The two patients were comparable on the aspects of race, sex, and age at onset of primary ccRCC. Although the microenvironment plays an important role in evolutionary process of the metastatic cells from their primary tumors, the selected metastatic cancer cells maintain their characteristics following long-term *in vitro* cultures. *In vivo* study demonstrated that MRCC cells exhibited more malignant and metastatic potential than NRCC (Table 2). Therefore, the differences in cellular and subcellular morphology, cell growth/invasion ability, cytogenetics, cell markers, and expression pattern of metastasis-associated molecules between the two cell lines can designate, at least partially, some important cellular and molecular events related to ccRCC metastasis.

The current study characterized the cell lines of 50–60 generations. It was found that MRCC grew a little slower but exhibited stronger anchorage-independent growth potential than NRCC *in vitro.* The invasion study indicated MRCC had higher invasion potential than NRCC. Analysis of cell cycle at the same pace of proliferation suggested that MRCC were more frequent in S phase whereas NRCC displayed a typical sub-G1 peak. Thus, compared to NRCC, MRCC exhibited “low proliferation-high invasion-low apoptosis” cell kinetic profile. This is probably related to a large number of glycogen particles stored in the cytoplasm. The “glassy” cytoplasm appearance of ccRCC might be due to glycogen and sterol storage caused by abnormalities in carbohydrate and lipid metabolism [9,11]. GSK3 is a protein kinase that phosphorylates and inactivates glycogen synthase, the final enzyme of glycogen biosynthesis [12]. In this study, we found that GSK3β, an important member of GSK3 family, was higher expressed in NRCC than in MRCC (Figure 6). The level of GSK3β may be reversely related to glycogen storage in RCC cells. Glycogen-rich carcinomas of clear cell subtype are usually characterized by a peculiar “low proliferation-low apoptosis” cell kinetic profile and associated with cancer aggressiveness [11,13]. Thus, the level of GSK3β might be reversely related to ccRCC metastasis.

Loss of chromosomal materials on 3p, 8p, 9p, and 14q has been documented in 96%, 22%, 33%, and 41% of ccRCC cases, respectively [14]. The von Hippel Lindau (*VHL*) tumor suppressor gene on chromosome 3p and stabilization of HIF1α due to loss of VHL function has been shown to be central to development of ccRCC [15]. However, the cytogenetic abnormalities on chromosomes 3p and the difference in HIF1α levels were not found in this study. With the use of single-cell exome sequencing, *AHNAK, LRRK2, SRGAP3, and USP6* have been found to be the key mutated genes in the ccRCC patient without *VHL* mutations [16]. In this study, although we found some differences in their expression patterns (Figure 6), it is hard to interpret the role of the 4 genes in ccRCC in terms of the expression patterns. We found that gains of chromosomes and some abnormal structures were the major chromosomal abnormalities in the two cell strains. Thus, our findings add novel information to the cytogenetic abnormality of ccRCC with different metastatic potentials and make the cell lines good tools to study RCC without *VHL* mutations.

Our cytometry assay revealed that the two cell lines were positive for CD44 but negative for CD133, CD105, and CD74. CD44 is a hyaluronic acid receptor whose mRNA levels in tumors can distinguish between RCC subtypes and RCC subtypes from oncocytoma and predict RCC metastasis [17]. Unexpectedly, the two cell lines were negative for CD105, a possible marker of ccRCC-initiation cells [10]. Recent studies have confirmed that CD133 is not detectable in RCC cells and tissues [10,18]. Here we found CD24 positivity was more frequent in MRCC than in NRCC and the same was true for CD56 (Figure 4). CD24 is a cancer stem-like cell biomarker whose expression in tumors is associated with malignant phenotype and poor prognosis of ccRCC and other cancers [19,20]. CD56 has been found to be expressed in 15%-18% of ccRCC and associated with poor outcome [21]. Although no markers, single or combined, could be defined unequivocally to specifically identify cancer stem cells in a given solid tumor so far [22], our data indicate that CD24-positive subpopulation might be the most likely stem-like cells that are related to ccRCC metastasis. NRCC and MRCC are epithelial-origin, but MRCC tends to be more mesenchymal-like (Figure 4). Acquisition of mesenchymal properties by epithelial cells, a process called epithelial-mesenchymal transition (EMT), can partially explain the metastatic potential of MRCC.

Although TNFα, VEGF, IL-6 and other cytokine/chemokine from lymphocytes, endothelial cells and mesenchymal cells within the microenvironment are necessary to maintain cancer “stemness” [23], the expression of these factors in cancer cells is important in maintaining their invasive and metastatic potential. In this study, we found that the transcriptional levels of *IL-6*, *VEGF*, *HIF2α*, *TNFα*, *MMP2*, and *RhoC* were higher in MRCC than in NRCC. Expression of *VEGF*, *MMP2*, and *RhoC* in ccRCC is associated with metastasis and poor prognosis or used to evaluate the effectiveness of therapies on metastatic RCC [24-26]. The expression of *IL-6* and *TNFα* is significantly elevated in high malignant RCC cells compared to low malignant RCC cells [27]. Furthermore, plasma levels of TNFα and IL-6 are associated with poor survival of RCC patients [28]. Interestingly, the expression of HIF2α, rather than HIF1α, was significantly elevated in MRCC than in NRCC (Figure 6). The HIFα subunits increase target gene transcription in hypoxic cells. However, HIF1α uniquely activates glycolytic enzyme genes, while HIF2α preferentially activates VEGF and cyclin D1. HIF2α promotes while HIF1α inhibits *c-Myc* transcriptional activity and cell cycle progression in RCC [29]. HIF1α negatively regulates Wnt/β-catenin signaling, while HIF2α is required for β-catenin activation in RCC cells and for RCC proliferation [30]. HIF1α/HIF2α imbalance in cancer cells might be important for RCC growth and metastasis.

The expression patterns of *IL-6* and *TNFα* indicate that NF-κB signaling pathway is more active in MRCC than in NRCC. This result was later confirmed by the western blot findings that IκBα was more degraded in MRCC than in NRCC following the treatment with TNFα (Figure 7). Thus, NF-κB is not only critical in regulating RCC biology that pose challenge to conventional therapy [31], but also important in promoting ccRCC metastasis.

## Conclusions

We established two ccRCC cell lines named MRCC and NRCC from clinical specimens of two Chinese patients. ccRCC cells from metastatic tumor have more malignant and metastatic potential than those from primary tumor following long-term *in vitro* cultures. CD24 positivity and the transcription levels of pro-angiogenic, pro-inflammatory and growth signaling factors in cancer cells are associated with ccRCC invasiveness and metastasis. These comparable cell lines will be powerful tools to improve our knowledge about ccRCC biology and metastasis.

## Methods

### Clinical specimens and primary culture

A 62-year-old male patient (No.375771) underwent surgical resection for a growing lesion in the spine at the 2nd affiliated hospital on February 6, 2006. This patient had received nephrectomy to excise ccRCC 10 years ago. He was pathologically diagnosed as ccRCC metastasized to bone and tumor nuclear grade was Fuhrman III. A 49-year-old male patient (No.378570) underwent nephrectomy at the same hospital on April 3, 2006. This patient was histopathologically diagnosed as ccRCC. The tumor nuclear grade was Fuhrman II. The fresh surgical specimens were immediately transported to our laboratory in ice-cold PBS, and processed for cell culture within 60 min after surgery. Primary cell culture was performed as previously described [32]. The experimental protocol was approved by the Institutional Ethical Review Board of Second Minitary Medical University conformed to the ethical guidlines of the 1975 Declaration of Helsinki. An informed consent was obtained from each patient. The two patients were followed since receiving the surgery in our hospital. The patient with metastatic ccRCC died of ccRCC six months after the last surgery. The patient with primary ccRCC was radiographically diagnosed as having a tumor in pancreas in 2008 and died in 2009. However, we were not sure if the tumor was pancreas-originated cancer or metastasis from original ccRCC because this patient didn’t receive further surgery or biopsy.

### Morphology and electron microscopy (EM)

Morphology of the cells and their parental tissues was observed using inverted phase-contrast microscope (Leica DMI 3000B, Germany) following routine H&E staining as previously described [32]. Cells (5 × 10^6^) were processed as previously described [9] and examined using an electron microscope (Hitachi H-7650, Tokyo, Japan).

### Cell growth and invasion assay

A total number of 3 × 10^4^ cells for each cell line suspended in 12 ml DMEM (GIBCO, Grand Island, NY) with 10% FCS (GIBCO) were plated in 24-well plates. Cells in every three wells were counted once a day. The average numbers were used to generate the growth curve. Anchorage independent growth potential was evaluated by double-layered soft agarose culture system. After cultured for 15d, the cells were stained with crystal violet and colony formation was counted under a light microscope (Leica). Cell invasion assay was performed using 24-well tissue culture plates (8-μm pore size, Transwell, Corning, NY). The bottom of the culture inserts was coated with 20 μg of Matrigel (BD Biosciences, Bedford, MA). The cells (5 × 10^4^) in 0.1 ml medium with 1% FCS were placed in the upper chamber and the lower chamber was loaded with 0.2 ml medium containing 10% FCS. After cultured for 24 h in 37°C, 5% CO_2_, the cells that migrated to the lower surface of filters was quantified by counting 10 independent symmetrical visual fields under the microscope to determine the invasion rate. Each assay was performed in triplicate.

### Karyotype analysis

Chromosomal preparation and R-banding were performed as previously described [33]. A total of 100 metaphase spreads were observed under a microscope (Leica, DM6000B) and 30 complete karyotypes were prepared to determine the chromosome number of each cell line using CW4000 software (Leica).

### Flow cytometry

Cell markers were determined using the following monoclonal antibodies: anti-CD44-Phycoerythrin (PE), anti-CD74-PE, anti-CD105-FITC, anti-CD56-FITC, anti-CD24-FITC, and anti-CD99-FITC (Biolegend, UK); anti-CD133-PE (Miltenyi, Germany); anti-vimentin (Santa Cruz, CA); anti-N-Cadherin (BD Biosciences); anti-E-cadherin, anti-EpCAM (Cell Signaling, MA). Briefly, both cell lines were cultured at the same condition. Then 5 × 10^5^ cells were washed twice and resuspended in PBS with 1% FCS, and incubated either in the primary antibody (anti-CD133, CD44, CD74, CD105, CD56, CD24, and CD99) conjugated with FITC or PE for 30 min on ice, or incubated in primary antibodies to vimentin, E-cadherin, N-cadherin, or EpCAM and then washed and incubated with secondary antibody conjugated with FITC. Cells were then washed twice with PBS containing 1% FCS. Cell fluorescence was analyzed within 1 h using a flow cytometer (FACSCalibur, BD Biosciences). Cell debris and fixation artifacts were excluded by appropriate gating. The acquisition process was stopped when 10,000 events were collected in the population gate. CellQuest software (BD Biosciences) was used for data acquisition and analysis.

The cells for cell cycle analysis were grown at the same pace and fixed with 70% ethanol at 4°C for more than 2 h and then washed twice. Fixed cells were stained with 100 mg/ml propidium iodide containing 100 mg/ml RNase. Samples on ice were immediately analyzed on the flow cytometer with CellQuest software to separate G0/G1, S, and G2/M phases.

### Quantitative RT-PCR

The cells were cultured under the same condition in 6-well plates. Total RNA was isolated and reverse transcribed to cDNA, and subjected for quantitative PCR as previously described [34]. The assay for each gene was repeated for 4–5 times. The primers for the amplification of *IL-6*, *TNFα*, *VEGF*, *MMP2*, *HIF1α*, *HIF2α*, *GSK3β*, *RhoC, USP6, AHNAK, LRRK2,* and *SRGAP3* as well as their corresponding amplified conditions are summarized in Table 3, *GAPDH*, and *β*_*2*_*-microglobulin* were used as internal control.

**Table 3:** Primers and PCR amplification condition

**Genes**	**Primers**		**PCR condition**
	**Sense (5**^**′**^**-3**^**′**^**)**	**Anti-Sense (5**^**′**^**-3**^**′**^**)**	
*IL-6*	GCTTTAAGGAGTTCCTGC	GGTAAGCCTACACTTTCCA	95°C for 10 min. 45 cycles of 95°C for 10 s, 60°C for 10s, and 72°C for 25 s
*TNFα*	GTAGCCCATGTTGTAGCA	CTCGGCAAAGTCGAGATA	
*VEGF*	ACTGCTGTGGACTTGAG	CAGGTGAGAGTAAGCGA	
*MMP2*	GCAAGTTTCCATTCCGC	GTCGTCATCGTAGTTGGC	
*GAPDH*	GACCCCTTCATTGACCTCAAC	CTTCTCCATGGTGGTGAAGA	
*HIF1α*	GTTTACTAAAGGACAAGTCACC	TTCTGTTTGTTGAAGGGAG	95°C for 15 min, 40 cycles of 95°C for 10s, 60°C for 45 s
*HIF2α*	GTCACCAGAACTTGTGC	CAAAGATGCTGTTCATGG	
*GSK3β*	CTAAGGATTCGTCAGGAACAG	TTGAGTGGTGAAGTTGAAGAG	94°C for 3 min, 40 cycles of 94°C for 15 s, 60°C for 30s, 72°C for 30s
*RhoC*	TCCTCATCGTCTTCAGCAAG	GAGGATGACATCAGTGTCCG	30 cycles of 94°C for 30s, 58°C for 1 min,72°C for 1 min
*β*_*2*_*-microglobulin*	ACCCCCACTGAAAAAGATGA	ATCTTCAAACCTCCATGATG	
*USP6*	TCAGAAGAGTGTTGCCCCAT	GGCTTTTCATGGACTCGGTT	95°C for 3 min, 30 cycles of 94°C for 30s, 58°C for 1 min, 72°C for 1 min
*SRGAP*	GGATTCCCGAAGTGACAAGC	GACTGCAGCTGGTGATAACG	
*LRRK2*	TGGGTTGGTCACTTCTGTGC	CATTGGCTGGAAATGAGTGC	
*AHNAK*	GTGCCACCATCTACTTTGACA	GCTGGCTTCCTTCTGTTTGT	

### Western blot

The cells were *in vitro* treated with 10 ng/mL TNFα (R & D Systems, MN) at different time points and then harvested. Cytosolic protein extracts were prepared as previously described [35]. Cytosolic IκBα was determined by immunoblotting with an anti-IκBα antibody (Cell Signaling). β-actin was detected by immunoblotting with antibody against β-actin (Cell Signaling). Genetools software (version 4.02, Synoptics, Cambridge, England) was used to quantify the signal strength of the bands.

### Subcutaneous and orthotopic transplantation

Six-week-old male BALB/c nude mice were purchased from Shanghai Experimental Animal Centre, Chinese Academy of Science (Shanghai, China) and treated in accordance with the American Association for the Accreditation of Laboratory Animal Care guidelines. The cells were washed and re-suspended in 200 μl PBS, and subcutaneously injected into the flanks of the mice (2 × 10^6^/mouse). The mice were sacrificed when tumors grown up to 10 mm in diameter. The tumors were excised and mechanically minced with scissors in a sterile manner. Half of the minced tissues were subjected for primary culture, another half of the minced tumors of 1-2 mm in diameter were transplanted into renal subcapsules of anaesthetized mice to establish SOI model as previously described [36]. The mice were sacrificed before dying. The tumor tissues were transplanted for the next round of SOI. All visceral organs were fixed in 10% formalin. Metastasis was confirmed using gross and histological examination.

### Statistical analysis

Student’s *t* test was used to determine the differences in the colony-formation rates, invasion rates, and gene expression levels of the two cell lines. All statistical tests were two-sided and performed using the Statistical Program for Social Sciences (SPSS16.0 for Windows, Chicago, IL). A *p* value of <0.05 was considered as statistically significant.

## Abbreviations

RCC: Renal cell carcinoma; ccRCC: Clear cell renal cell carcinoma; EM: Electron microscopy; PE: Phycoerythrin; FITC: Fluorescein isothiocyanate; qRT-PCR: Quantitative reverse transcription-PCR; EMT: Epithelial- mesenchymal transition; GSK3β: Glycogen synthase kinase 3 beta; HIF: Hypoxia-inducible factor; IL-6: Interleukin-6; IκBα: Inhibitor of NF-κB; MMP2: Matrix metalloproteinase-2; NF-κB: Nuclear factor kappa B; SOI: Surgical orthotopic implantation; TNFα: Tumor necrosis factor-alpha; VEGF: Vascular endothelial growth factor; VHL: Von Hippel Lindau; USP6: Ubiquitin specific peptidase 6; AHNAK: AHNAK nucleoprotein; LRRK2: Leucine-rich repeat kinase 2; SRGAP3: SLIT-ROBO Rho GTPase activating protein 3.

## Competing interests

The authors of this paper have no potential conflict of interest to disclose.

## Authors’ contributions

XT analyzed whole data and drafted the manuscript. YH carried out chromosome analysis. SH was responsible for setting up animal model. ST was responsible for cell culture. YY was responsible for pathological analysis. JH, JR and DX were involved in the diagnosis and the recruitment of the patients in our affiliated hospitals. GW was responsible for statistical analysis. YD and JY revised it critically for important intellectual content; GC designed and organized the study and revised the manuscript. All authors have read and approved the final manuscript.
